# A Lightweight Method for Detecting IC Wire Bonding Defects in X-ray Images

**DOI:** 10.3390/mi14061119

**Published:** 2023-05-26

**Authors:** Daohua Zhan, Jian Lin, Xiuding Yang, Renbin Huang, Kunran Yi, Maoling Liu, Hehui Zheng, Jingang Xiong, Nian Cai, Han Wang, Baojun Qiu

**Affiliations:** 1 State Key Laboratory of Precision Electronic Manufacturing Technology and Equipment, Guangzhou 510006, China; zhandaohua@mail2.gdut.edu.cn (D.Z.); linjian7794@163.com (J.L.); 2112101016@mail2.gdut.edu.cn (X.Y.); hrenbin249@163.com (R.H.); kunranyi@163.com (K.Y.); liumaolingdut2020@163.com (M.L.); 2112001294@mail2.gdut.edu.cn (H.Z.); 2112001309@mail2.gdut.edu.cn (J.X.); cainian@gdut.edu.cn (N.C.); 2 School of Electromechanical Engineering, Guangdong University of Technology, Guangzhou 510006, China; 3 School of Information Engineering, Guangdong University of Technology, Guangzhou 510006, China; 4 China Electronic Product Reliability and Environmental Testing Research Institute, Guangzhou 511370, China

**Keywords:** convolutional neural network, X-ray images, wire bonding defects, lightweight network

## Abstract

Integrated circuit (IC) X-ray wire bonding image inspections are crucial for ensuring the quality of packaged products. However, detecting defects in IC chips can be challenging due to the slow defect detection speed and the high energy consumption of the available models. In this paper, we propose a new convolutional neural network (CNN)-based framework for detecting wire bonding defects in IC chip images. This framework incorporates a Spatial Convolution Attention (SCA) module to integrate multi-scale features and assign adaptive weights to each feature source. We also designed a lightweight network, called the Light and Mobile Network (LMNet), using the SCA module to enhance the framework’s practicality in the industry. The experimental results demonstrate that the LMNet achieves a satisfactory balance between performance and consumption. Specifically, the network achieved a mean average precision ($mAP_{50}$) of 99.2, with 1.5 giga floating-point operations (GFLOPs) and 108.7 frames per second (FPS), in wire bonding defect detection.

## 1. Introduction

Integrated circuits are an indispensable core component in electronic products such as mobile phones, smart watches, computers, and intelligent robots [1,2,3]. Integrated circuit design, packaging and testing are the three pillars of integrated circuit manufacturing, and IC packaging is one of the important factors restricting the development of this field [4,5]. Packaging costs and precision directly determine the cost of IC product manufacturing, and wire bonding [6,7,8] is one of the most important steps in IC packaging technology; it uses ultrasonic, pressure, heat, and other energy forms to connect the internal pins of the IC chip and the pins of the external substrate, or the pins between the lead frames, which determines the quality and stability of power supply and signal transmission. However, during the wire bonding process, various defects may occur due to certain problems, such as depression of the solder joint, cracking and peeling of the bonding, etc., which lead to the failure of the wire bonding. In addition, different factors, such as human error, material selection, processing equipment, manufacturing process, etc., affect the bonding process, making the determination of wire bonding defects challenging. In order to reduce the costs of integrated circuit manufacturing and improve the yield of IC chips, it is very important to accurately assess the quality of IC wire bonding after packaging. In today’s context of the large-scale production of integrated circuits, fast, automatic, and accurate detection of wire bond defects is highly desired in relation to integrated circuit quality and cost control.

X-ray imaging is widely recognized as a cost-effective inspection method in industrial defect detection [9,10], quality control, and safety inspection. For identifying defects hidden inside the product that cannot be captured by cameras, non-destructive testing methods, such as X-rays, are typically used. In the actual production of IC chips, wire bonding defects shown by X-ray images are still manually inspected, which has low efficiency, is prone to fatigue-related problems, and is subject to differences in perception and emotion, making it unable to meet the current mass production requirements of IC chips. Moreover, manual inspection is also costly. Therefore, to optimize the potential of image recognition, and realize automatic IC image analysis and wire bonding defect detection, we aimed to identify defect information via carrying out an object detection task.

**Figure 1:**
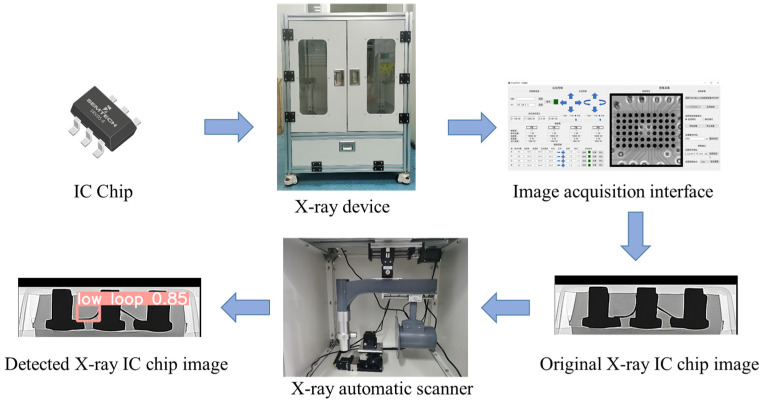
The inspection system of an X-ray IC chip image, which comprises two stages. In the first stage, a batch of IC chip images is collected and used to train our network. In the second stage, the trained model is applied to the remaining IC chips for real-time defect detection.

Figure 1 shows a flowchart of the IC chip image detection system. To better understand the acquisition and identification of IC chip wire bonding defects, we sought to obtain images of the wire connections inside the chip using X-ray imaging. We built our X-ray equipment to obtain inside images of IC chips. In the side view of the chip, two wires are visible, and this is a direct reflection of the quality of the chip’s wire bonding. There are typically five types of wire bonding defects: high loop, low loop, broken line, defect, and vertical line. The appearance of these defects can affect the performance of the IC chip and thus represented the target of model inspection.

In recent years, deep learning methods have undergone a surge in popularity, with convolutional neural network (CNN)-based methods achieving exceptional results in various imaging-related tasks [11,12,13]. Kyeong et al. [14] proposed building a classification model for each hybrid defect model in the semiconductor manufacturing industry to detect on-chip circles, rings, scratches, and regional defects. However, creating a separate model for each defect increases computer power consumption. Mao et al. [15] proposed using a convolutional neural network to analyze the IC dataset and optimize the VGG16 network to detect IC defects. However, this method requires a large amount of data and involves a long training time. Chen et al. [16] developed an adaptive deep learning framework for fast marker recognition in IC chips, but this has limited adaptation capabilities and cannot detect the same types of defects across large scale changes. Yang et al. [17] proposed using the YOLOv3-based network to detect chip defects and fine-tune the network, resulting in significant improvements in detection accuracy, with the $mAP_{50}$ reaching 86.36. However, this network requires much model calculation and has many parameters, which makes it unsuitable for deployment in industrial computers or mobile devices. Chen et al. [18] proposed a data-driven framework for detecting wire bonding defects in IC chips, but the method is highly reliant on environmental variables and may be adversely affected by changes in lighting conditions and chip location area, leading to poor segmentation and defect recognition. Zhou et al. [19] described the development history of wire bonding, and the manufacturability and reliability of gold, copper, and silver wire bonding. They also discussed the basic performance and gave a general comparison of applications among the three types of wire bonds. Kao et al. [20] developed a deep-learning-based fault diagnosis framework that can effectively detect improper bond head installation in wire bonding equipment, enabling predictive maintenance and reducing costs. None of the previous studies have found an optimal solution for detecting IC chip wire bonding defects, which involves achieving a balance between inference speed and model energy consumption. Therefore, further exploration in the field of deep learning is necessary for the detection of IC chip wire bonding defects.

In this paper, we present a lightweight and efficient X-ray-image-based wire bond defect detection framework for IC chips, which uses industrial CT equipment consisting of an X-ray tube, a flat panel detector, a four-axis motion platform, and other components. Our framework includes a lighter, and mobile, YOLO network (LMNet) for wire bond defect identification, inspired by the efficient structure of YOLOv5n and EfficientNet [21]. We propose a lighter LMNet based on a new Spatial Convolutional Attention (SCA) module, which integrates multi-scale features and adaptively weights them according to their spatial and channel contributions. Compared with previous studies, this proposed chip inspection method aims to solve the problems of slow detection speed and high energy consumption associated with identifying wire bonding defects in IC chips after packaging.

To validate the effectiveness of our framework, we conducted extensive experiments on our dataset. The experimental results demonstrate that our framework is faster and involves fewer parameters than the current state-of-the-art methods. The organization of this paper is as follows: Section 2 describes the collected data and associated methods. Section 3 presents our proposed method. Section 4 outlines the experimental results and analyzes them.

In summary, this work makes the following contributions:

- Fully automatic industrial CT equipment was built for IC chip image recognition and acquisition;
- A lightweight and precise frame LMNet was designed to realize automatic, fast and high-performance IC chip wire bonding image recognition;
- We proposed to introduce an adaptive Spatial Convolutional Attention (SCA) module into LMNet to achieve adaptive weighting of multi-scale features;
- The experimental results demonstrated that our method achieves fast and accurate prediction compared to state-of-the-art models.

## 2. Related Works

### 2.1. Experimental Setup

**Figure 2:**
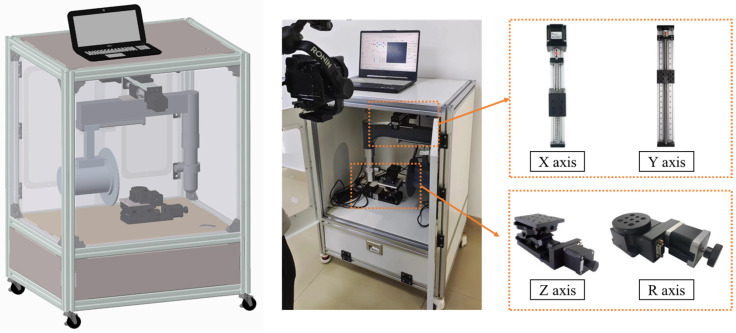
The 3D structure and a physical drawing of the experimental X-ray chip detection device.

Our experimental equipment for X-ray chip detection consisted of several components, including an X-ray source system, a flat panel detector acquisition system, a four-axis precision motion platform, a computer-aided system, and an X-ray protection device. These hardware components were integrated and connected in series to the industrial computer, which read the image collected by the flat panel detector through the data acquisition card. The acquisition card and detector were connected via an AV signal, while the industrial computer and acquisition card were connected via USB. The motion controller was connected to the industrial computer via Ethernet, and the controller was connected to the four-axis drive motor to enable the motion control of each axis. Finally, the industrial computer was connected to the embedded control circuit via the RS485 serial port to control the emission and power of the X-ray. Figure 2 shows the 3D structure and gives a physical diagram of our experimental device.

### 2.2. Data Acquisition

**Figure 3:**
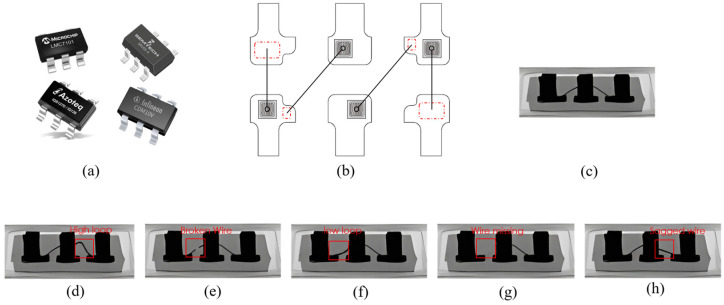
(**a**) The targeted IC chip; (**b**) schematic diagram of the chip; (**c**–**h**) illustrative examples of IC wire bonding; (**c**) normal bond wire; (**d**) high loop wire; (**e**) broken wire; (**f**) low loop wire; (**g**) missing wire; and (**h**) sagged wire.

We utilized self-built X-ray equipment for image acquisition and processing, comprising an X-ray source, a flat panel detector, a motion control card, and a four-axis motion platform, with parameters listed in detail in Table 1. Real-time defect detection was performed using a portable notebook configured with an AMD R7-5800CPU, 16 G RAM, and an Nvidia RTX3060 graphics card. To facilitate the use of the equipment by other computers, automatic IC chip detection software was developed for the Windows platform, with the acquisition of IC chip datasets. The integrated circuit product used in this paper is shown in Figure 3; the X-ray image taken from the side view shows the two wires, shown in Figure 3b. An example of a qualified IC chip is illustrated in Figure 3c, while the five types of welding line defects shown in Figure 3d–h, namely, high loop line, low loop line, broken line, defect, and vertical line, are the targets of the detection model in this paper. We collected 800 IC chip images using our self-developed software, which were adjusted to a size of 416 × 416 pixels for network training. Data augmentation was performed through a combination of rotation, random cropping, color channel transformation, and Gaussian blurring, with each image randomly enhanced twice, resulting in a total of 1600 images. These images were randomly divided into a training set, a validation set, and a test set in a ratio of 8:1:1. Table 2 shows the quantity distribution of the sample dataset in this paper.

### 2.3. Object Detection

To date, there are two main types of deep learning algorithms used for target detection: single-stage detectors based on regression analysis and two-stage detectors based on region proposal. Traditional two-stage detectors, such as Rfi-CNN [22], first use selective searching to extract potential target regions, then employ a CNN to extract features from these candidate regions after adjusting their size. This is followed by the use of a support vector machine classifier to classify the region, and the location information is obtained through fully connected neural network regression. However, the R-CNN network has problems, such as the requirement of independent training at each stage, the cumbersome nature of training, susceptibility to image distortion, high computational power consumption, and slow detection speed. To address these issues, Faster R-CNN [23] involves a region proposal network (RPN) to replace the selective search algorithm in time-consuming candidate region extraction. The RPN in this network incorporates a multi-reference window mechanism, which completes candidate region recommendation, feature extraction, localization, and classification in the same network, greatly improving training efficiency. However, due to the anchor mechanism, Faster R-CNN’s detection accuracy with small targets is not ideal. Mask R-CNN incorporates the Mask branch, and combines the image semantic segmentation and object detection network. By adding a linear interpolation algorithm to prevent the feature map and original image from deviating due to the integer quantization of the ROI pool, the features obtained from each receptive field can be made to more effectively align with the original image’s receptive field area. However, Mask R-CNN [24] has a larger computational overhead than Faster R-CNN due to the segmentation branch. Libra R-CNN [25] includes IoU-balanced sampling, a balanced feature pyramid structure, and a balanced L1 loss function, thus effectively solving the problem of features disappearing after small samples pass through a multi-layer network. However, the Libra R-CNN network is stacked, structurally redundant, and has a large number of parameters, resulting in a slow detection speed. Although two-stage detectors can usually obtain more accurate prediction results, they require more computing resources, and their detection speed is not ideal.

Single-stage detectors are better suited for tasks requiring fast inference, and their main advantage over two-stage detectors is the absence of a candidate region recommendation stage, resulting in a simpler training process. In a single stage, the target category can be directly determined and the position detection frame can be obtained. The YOLO [26] algorithm was the first single-stage detector to be used in deep learning, and uses a single neural network to divide the entire image into S × S network units, thus determining whether the predicted target center falls within the network. The grid then determines the prediction object category and corresponding confidence, followed by threshold screening to remove windows with low target probability and an NMS to remove redundant windows, improving inference speed. However, YOLO can only detect one bounding box with the highest IoU output, resulting in only one detection for multiple small targets in a grid. Additionally, YOLO does not address the multi-scale window problem, resulting in poor small-target detection and inaccurate positioning compared to Faster R-CNN. SSD [27] combines the advantages of the fast detection of YOLO and the accurate positioning of Faster R-CNN, and introduces multi-reference and multi-resolution detection technology, whereby the networks of different layers detect objects with different scales, which effectively improves the detection of small objects. However, the basic size and shape of the pre-selection box in the network need to be manually set; the debugging process is highly dependent on experience, the recognition effect of small targets is general, and the feature extraction is insufficient. RetinaNet [28] achieves a further improvement in the detection accuracy of integrated convolutional neural networks by introducing a focal loss function that prioritizes difficult-to-classify samples during training to solve the problem of unbalanced instance samples. However, single-stage detectors are less accurate than two-stage detectors in most cases due to the lack of region proposals.

To improve the practicality of CNN, efficient methods can be developed to design new network structures, such as lightweight convolutional neural network models. SqueezeNet [29] includes a Fire Module, which comprises Squeeze and Expand layers that help to reduce the dimension of the input feature channel and achieve feature extraction. MobileNet [30] uses depthwise separable convolution to design basic building blocks, and can be easily adjusted with two hyperparameters to reduce model complexity. ShuffleNet [31] represents an improvement of ResNet’s residual unit, with grouped convolution and channel shuffling to reduce the number of model parameters and enable information exchange between different groups. GhostNet [32] introduces a Ghost Module that generates feature maps inexpensively, thus effectively addressing feature map redundancy in convolutional neural networks and reducing the number of parameters.

### 2.4. Multi-Scale Features Fusion

In the task of target detection, accurately identifying and precisely positioning targets can be challenging due to the varying shapes and sizes of objects. Feature fusion has been proven to be an effective strategy for achieving feature complementarity between different layers of the CNN. However, early fusion methods, such as simply adding or concatenating multi-scale features, may lead to significant losses of detailed features. To address this, SSD [27] and MS-CNN [33] propose the separate detection of objects on feature maps of different scales, and integrating them in the end, with shallow feature maps detecting small objects and deep feature maps detecting large ones. However, these methods have not significantly improved the detection accuracy of small targets. To tackle this issue, FPN [34] combines the fine-grained spatial information of shallow feature maps and the semantic information of deep feature maps to construct a top-down structure for multi-scale targets. In recent years, it has been found that multi-scale fusion based on different receptive fields can greatly improve CNN performance. For instance, the SPP module proposed in SPP-Net [35] uses multi-scale blocks to convert feature maps of any size into fixed-length feature vectors. Similarly, ASPP [36] builds a spatial 16 pyramid using atrous convolution with different convolution coefficients, resulting in multiple sets of feature maps. However, contemporary feature fusion methods treat all scales equally, and cannot adaptively consider which scale features are more important for the final prediction.

## 3. Method

### 3.1. Overview of YOLOv5

YOLOv5 consists of five models, namely, YOLOv5n, YOLOv5s, YOLOv5m, YOLOv5l, and YOLOv5x. YOLOv5 adopts the C3 architecture, with SPPF as the backbone layer, PANet as the neck layer, and a YOLO detection head, making it the fastest and most convenient single-stage detector. As our baseline network, we chose YOLOv5 for its performance. During training with the IC chip dataset, we observed that YOLOv5s yields similar results compared to the other models in the series, with an AP difference of less than 0.2. Since the YOLOv5s model incurs lower computational costs during training and inference, we chose it as our recognition network so as to strike a balance between detection speed and accuracy. Furthermore, we have proposed a new feature map fusion method called SCA to enhance the multi-scale recognition ability of the recognition network.

### 3.2. LMNet for IC Defect Recognition

**Figure 4:**
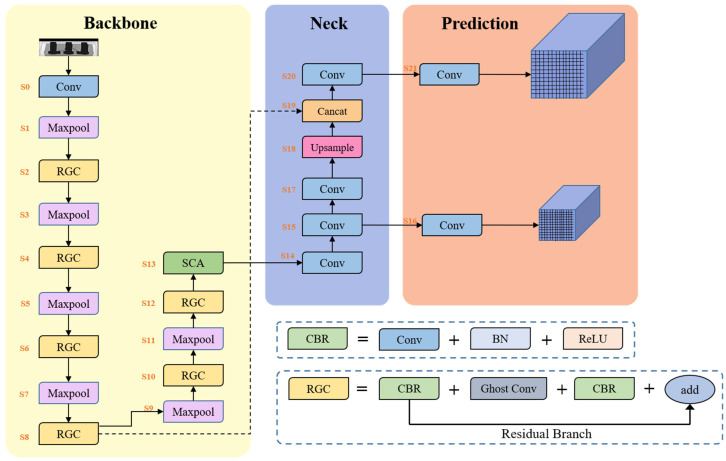
The architecture of the IC bond wire identification framework, which consists of LMNet for IC chip information identification. LMNet has two prediction heads that perform feature extraction on images and identify defects on X-ray IC chip images.

The LMNet framework, as shown in Figure 4, is a modified version of the original YOLOv5 network that is compressed and optimized for our IC defect dataset. In this section, we introduce two key components of the network—the Residual Ghost Convolution (RGC) module and the Spatial Convolution Attention (SCA) module—and provide a detailed description of the LMNet structure.

**Figure 5:**
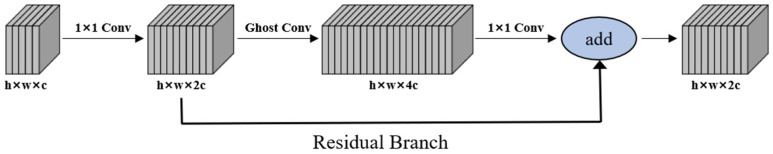
Illustration of our RGC module. This expands the weld feature in channel space to encode more implicit information, and Ghost Conv is used to reduce the module’s complexity.

RGC module: The Residual Ghost Convolution module is the basic module of a deep learning network. Efficient representation encoding can make the model better at its corresponding task. Furthermore, feature extraction operations are the main source of parameters and computations. Therefore, the weight of the feature extraction module determines the weight of the entire network. In this paper, we designed the RGC module as shown in Figure 5; this makes it lightweight, and it has high feature extraction capabilities in relation to the representation learning of X-ray IC images.

The RGC module consists of a 1 × 1 convolution, which increases the number of channels of the input feature map to 2c. To more effectively extract feature information, we needed to map the input data to higher-dimensional space in the intermediate stage. However, this can increase the computational load and memory consumption of the network. To address this issue, we have taken inspiration from GhostNet and introduced Ghost Conv into the feature space expansion process. Ghost Conv helps us to obtain more feature maps in an inexpensive way, thereby reducing memory consumption during intermediate expansion. Additionally, we introduced residual connections in the RGC module to ensure the effective extraction of feature information and improve the stability of the network.

Residual connections can effectively solve a range of problems caused by increases in the network depth, such as gradient disappearance, gradient explosion, and overfitting. We added residual connections to the RGC module to prevent the overfitting caused by the increase in the number of network layers, which effectively improved the stability of the network. Residual connections can be expressed as a superposition of input and nonlinear changes in the input. We defined the input and output of the lth layer as $X_{l}$ and $X_{l+1}$, respectively, and the nonlinear change of the input was defined as $F\left(x,w\right)$, where w represented the weight parameter of the function F. The residual connection calculation formula is expressed as follows:

(1) $$X_{l+1}=X_{l}+F\left(x_{l}+w\right)$$

Ghost Conv can avoid the redundant computation and convolution filters generated by similar intermediate feature maps, and can achieve a good balance between accuracy and compression. We defined the input feature map as $M\in {\mathbb{R}}^{h\times w\times c}$, where h, w, and c are the height, width, and number of channels of the input feature, respectively. The feature map of N could be generated through a convolution process:

(2) N=M⊗f

where ⊗ represents the convolution operation, $f\in {\mathbb{R}}^{c^{\prime }\times k\times s\times p}$ represents the convolution filter, and $c^{\prime }$, k, s, and p are the number of output channels, kernel size, stride and padding of filter f, respectively. The feature height, width, and number of channels of the output feature map $N\in {\mathbb{R}}^{h^{\prime }\times w^{\prime }\times c^{\prime }}$ were $h^{\prime }$, $w^{\prime }$, and $c^{\prime }$, respectively. To simplify the formula, we omitted the bias value.

However, in Ghost Conv, intrinsic feature maps are first generated using traditional convolution. Specifically, the intrinsic map $N^{\prime }\in {\mathbb{R}}^{h^{\prime \prime }\times w^{\prime \prime }\times c^{\prime \prime }}$ was generated using traditional convolution:

(3) $$N^{\prime }=M⊗f^{\prime }$$

where the convolution filter used is $f\in {\mathbb{R}}^{c^{\prime \prime }\times k\times s^{\prime }\times p^{\prime }}$. In order to keep the space size consistent with Equation (2), the height value $h^{\prime \prime }$ and the width value $w^{\prime \prime }$ remained unchanged. In order to obtain a feature map with the required number of channels $c^{\prime }$, Ghost Conv performed a cheap linear operation on each intrinsic feature to generate the required s ghost features, according to the following function:

(4) $$n_{ij}=\xi_{ij}\left(n_{i}^{\prime }\right),\forall i=1,2\ldots ,c^{\prime \prime },j=1,2,\ldots s$$

where ${n_{i}}^{\prime }$ is the *i*th intrinsic feature map of $N^{\prime }$, and $\xi_{i,j}$ is a linear operation to generate the *j*th ghost feature map $n_{i,j}$. Finally, we obtained $c^{\prime \prime }$ = $c^{\prime }s$. The output feature map was $N=\left[n_{11},n_{12},\ldots ,n_{c^{\prime \prime }s}\right]$.

The intermediate expansion stage of the RGC module doubles the output channel compared to the input channel, which effectively increases information retention through higher-dimensional feature maps. However, this operation may consume a significant amount of memory and require much computation. The introduction of Ghost Conv can greatly reduce this burden. The final 1 × 1 convolution reduced the channel count back to the original input dimension of 2c. To ensure network stability during training, we included the input of the expansion operation and the output of the second 1 × 1 convolution unit as a residual branch. This approach reduced network complexity while maintaining stability during training.

**Figure 6:**
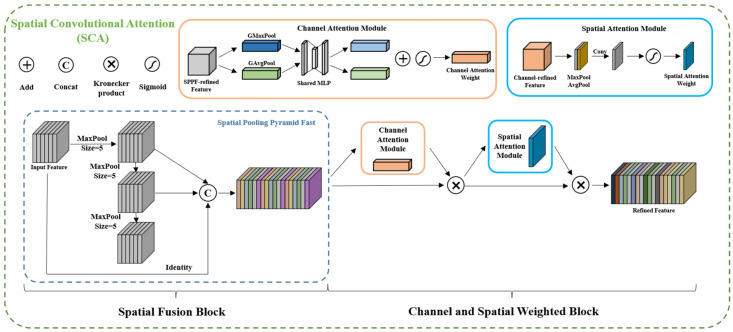
The internal structure of the proposed SCA module consists of a spatial fusion block and channel and a spatial weighted block, which enabled us to process the image successively. The first module is SPPF, which can quickly generate pooled pyramids for feature fusion. The second block is CBAM, which can determine the importance of spatial information for each channel.

**Figure 7:**
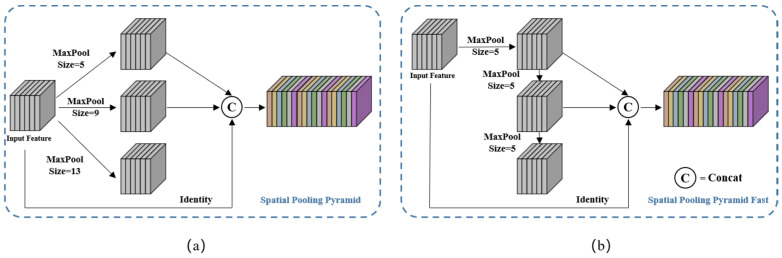
The structures of the SPP and SPPF modules are shown in (**a**,**b**), respectively. The SPPF module produced the same output as the SPP module, but with greater computational efficiency.

SCA module: To better utilize multi-scale features, we propose a novel SCA module, the structure of which is illustrated in Figure 6. SCA comprises two blocks, spatial scale fusion and attention weighting, and the feature maps are sequentially processed through these blocks. Spatial scale fusion was achieved using Spatial Pyramid Pooling (SPP), which mainly focused on spatial information and consists of four parallel branches: three max-pooling operations (with kernel sizes of 5 × 5, 9 × 9, and 13 × 13) and the input feature map itself. SPP effectively addresses the problem of excessive object scale variation by fusing local and global features. We used an improved version of SPP, called SPPF, based on the author’s work on YOLOv5. SPPF achieved an efficiency improvement of nearly 277.8% compared to SPP. The efficiency gain, $\eta_{c}$, was calculated using the following formula:

(5) $$\eta_{c}=\left[\left(k_{1}^{2}+k_{2}^{2}+k_{3}^{2}+\ldots +k_{i}^{2}-i\right)-\left(k_{1}^{2}-1\right)i\right]100\%$$

where $k_{i}$ is the kernel size of the i-th branch of max pooling in the SPPF module. Figure 7a,b illustrate the structures of the SPP and SPPF modules, respectively. 

The SPPF module was utilized in the spatial scale fusion part of the SCA module, while the attention mechanism module was used in the other part. The attention weighting block acted as an adaptive regulator that learned the importance of each channel’s spatial information, revealing which scale features are more prominent. Whereas multi-scale information is essential to developing effective feature maps, different scales may contribute differently to the results, especially when objects are of similar sizes. In such cases, only one scale may be critical for final prediction. The scale distribution of chip information was more consistent compared to other foreground contents. Therefore, the attention weighting block adaptively weighed different scales during network learning, giving greater weight to more meaningful scale features.

**Figure 8:**
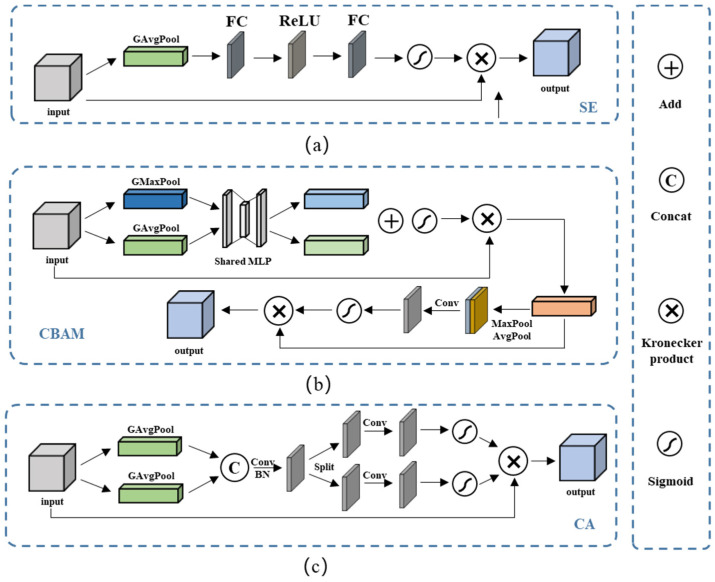
The structures of the three different attention modules. (**a**) The SE module, where the character “GAvgPool” means the global average pooling 2D layer, and the character “FC” means the fully connected layer. (**b**) The CABM module, where the character “Conv” means the ordinary convolution 2D layer. (**c**) The CA module, where the character “BN” means the batch normalization layer.

Currently, the most commonly used attention mechanisms are SE, CBAM, and CA modules. Among them, SE is a channel attention module that consists of two operations: squeezing and excitation. This module enables the network to focus on feature channels with greater informative content, while ignoring those with less information. On the other hand, CBAM is a spatial channel attention mechanism module that combines spatial and channel attention. The CA module is a novel approach that addresses the loss of location information caused by global pooling operations. By focusing on the width and height dimensions separately, the spatial coordinate information of the input feature map can be efficiently utilized. Figure 8 illustrates the structure of the SE, CBAM, and CA modules.

Overall, this paper proposes an SCA module, which integrates more information sources and adaptively weights them based on their importance, thereby improving the contextual representation ability of the feature map. Experimental comparisons show that the CBAM module achieved better results in SCA, and the role of SCA is discussed in detail in Section 4.1.

In terms of architecture, we drew inspiration from YOLOv5n and designed an IC chip defect recognition network called LMNet, which is shown in detail in Table 3. Compared with YOLOv5n, LMNet has fewer network layers and narrower models, resulting in reduced parameters and failures. To obtain a smaller bandwidth backbone, we strictly limited the number of channels in each layer, with almost all layers having fewer than 512 channels. This design strategy makes the network less computationally burdensome for devices. We embedded the RGC module in the backbone for deeper representation learning and efficient feature extraction, and the SCA module was positioned at the end of the backbone to ensure that it processed more meaningful information and could bring the enhanced features closer to the output layer for more accurate recognition results.

To train the LMNet model using the specific argmax shown in Algorithm 1, the resulting weights and features will be organized in .pt files. Subsequently, the LMNet structure and weights can be deployed on the device through the network connectivity.
**Algorithm 1. Proposed Lightweight Framework (LMNET)****Input:** I_x_ = X-ray image(input)**Parameters:** m = model, tr = train, v = valid, te = test, a = argmax (m_input-layer_, m_averagepooling2d_, m_flatten_, m_dense_, fl = feature-layer, α = learning-rate, es = early stopping, b = dataloader_batch, e = epoch), Ac= accuracy, image-shape = 640, class = Image-label.**Output:** Pci [P_class0_, …, P_class8_] Predicted class index**Preprocessing:**if set == training_set:xresize I_x_ to 512 × 512, crop and resize(I_x_) to 640, flipping, normalize pixel to (0, 1), pixels /= 255.0else: resize Ix to 640, normalize pixels to (0, 1), pixels /= 255.0**Models training:**for x = 1 to 300 [tr, v] = partition(tr, v)   for y = (tr/b, v/b)   if Ac_v_ < 99:    continue;    if Ac_v_ is not improving    for next 5 epochs, then    increase α = α x 0.1:      t(m, c, j) = train[m(c), a, tr(x), v(x)]       v(m, c, j) = valid[(m(c), a, x), v(x)]       unfreeze fl    else:      call es(m);   else:    break;  end y; end x;te(m(c), a) = [test(m(c), a, te)]Pci[P_class_] = te(m(c), a

## 4. Experiments

In this section, we present experimental results and their analysis to illustrate the superiority of our framework for use in IC chip defect identification. We first introduced the experimental setup, including implementation details and evaluation metrics. Ablation studies were then conducted to confirm the contributions of the RGC and SCA modules. Specifically, the ablation study aimed to demonstrate the necessity of the modules and visualize the weight values to demonstrate the weight distribution mechanism. Finally, for the defect identification task, we compared our proposed method with other state-of-the-art methods.

### 4.1. Experimental Setup

All models were implemented using the PyTorch deep learning framework. In the detection experiments of this study, the hyperparameters of the network were fine-tuned through a large number of experiments based on default parameters, and the optimal hyperparameters obtained were as follows: 100 epochs were trained, Stochastic Gradient Descent (SGD) was used as the optimizer, with a batch size of 16. A linear decay learning rate scheduling strategy was adopted, with an initial learning rate of 0.01 and a final learning rate of 0.001. The momentum parameter was set to 0.937 and the weight decay to 0.0005.

As regards the evaluation metrics, we used mean average precision at 50% intersection over union ($mAP_{50}$), recall rate (Recall), floating point operations (FLOPs), parameters (Params), and frames per second (FPS) to comprehensively evaluate the proposed network. $mAP_{50}$ and Recall were used to assess the detection performance, while the other metrics were used to evaluate computational complexity and speed. These metrics were defined as follows:

(6) $$Precision=\frac{TP}{\left(TP+FP\right)}$$

(7) $$recall=\frac{TP}{\left(TP+FN\right)}$$

The terms TP, FP, and FN represent important concepts in object detection, and are defined as follows:

- True Positives (TP)—The number of correctly detected objects;
- False Positives (FP)—The number of false detections, which include both absent and misplaced predictions;
- False Negatives (FN)—The number of objects that were not successfully detected by the model.

**Figure 9:**
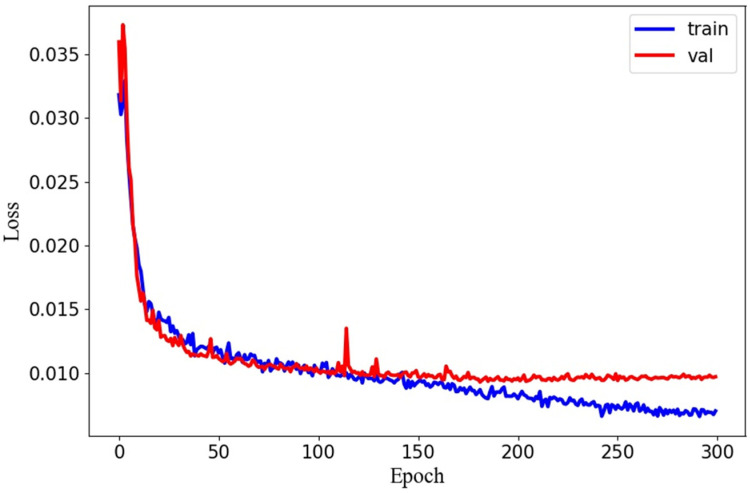
Losses occurred during training and the validation phase.

**Figure 10:**
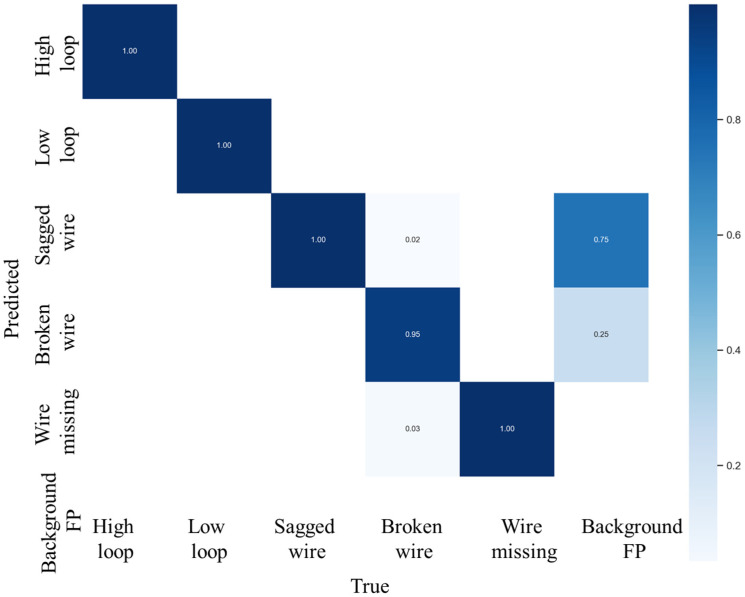
The confusion matrix for LMNet in the testing phase, which is presented with *x*- and *y*-axis labels ranging from 0 to 4, where 0 represents high loop, 1 represents low loop, 2 represents sagged wire, 3 represents broken wire, and 4 represents wire missing.

$mAP_{50}$ is an evaluation metric used to measure the overall performance of object detection across all categories. It is calculated as the average $AP_{50}$ value for all categories, where $AP_{50}$ is the area under the precision–recall curve. To compare the computational complexity of different networks, we used FLOPs to measure time complexity and Params to measure space complexity. During the inference stage, FPS was used to represent the speed of inference, which was calculated as the average of 160 test images. The loss functions for the dataset and validation set during the experiment are shown in Figure 9. In the data preparation stage, a confusion matrix was used to verify the classification performance of the model. This matrix compares the actual category with the predicted category, providing a more intuitive visualization of the model’s predictive performance. The confusion matrix of the model is shown in Figure 10.

### 4.2. Ablation Studies

Using LMNet as the base model, we conducted ablation studies on RGC and SCA modules, providing the theoretical foundation for this research. Specifically, we performed ablation studies on residual blocks and Ghost Conv in the RGC module. Table 4 shows that the RGC module achieved the best trade-off. When the Ghost Conv was replaced with the normal Conv, the $mAP_{50}$ dropped to 99.0, which was 0.2 lower than the full module. However, this design’s GFLOPs and Params were both higher than those of LMNet. Our LMNet outperformed other combinations in terms of performance, computation, and storage.

SCA is composed of SPPF and an attention mechanism. As shown in Table 5, the SE module within the attention mechanism had a greater impact on the GFLOPs and Params of the model. The number of parameters and computational cost of the CBAM and CA modules were not significantly different, but the $mAP_{50}$ of CBAM was 0.1 higher than that of CA. When the number of model parameters and the computational cost are comparable, accuracy is given more importance. Therefore, the attention mechanism in our designed SCA module utilizes CBAM.

### 4.3. Comparisons with the State of the Art

To evaluate the effectiveness of our proposed LMNet for IC chip defect detection, we compared our method with several state-of-the-art models, including the two-level networks Faster R-CNN and Dynamic R-CNN, and the one-level networks RetinaNet, SSD300, VFNet, YOLOv3, and YOLOv5. Additionally, we also conducted experiments by replacing the default backbone of YOLOv5s with lightweight backbones such as MobileNetV3, ShuffleNetv2, and GhostNet. The results of the quantitative comparison using our IC chip dataset are presented in Table 6.

**Figure 11:**
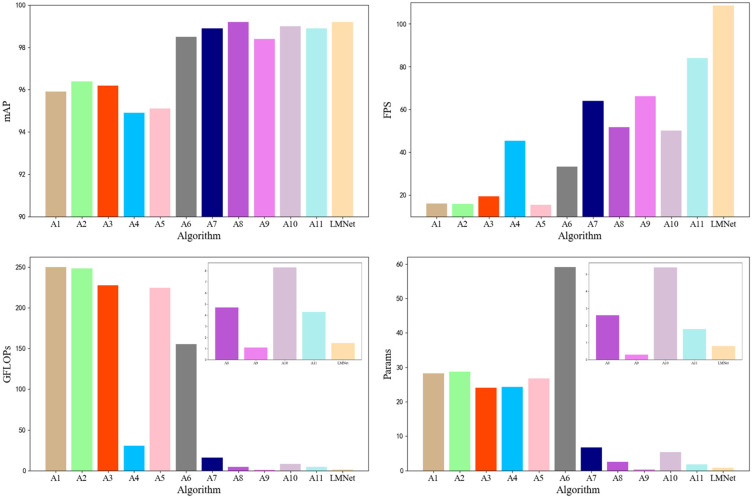
Bar chart of performance comparison between LMNet and other state-of-the-art networks.

**Figure 12:**
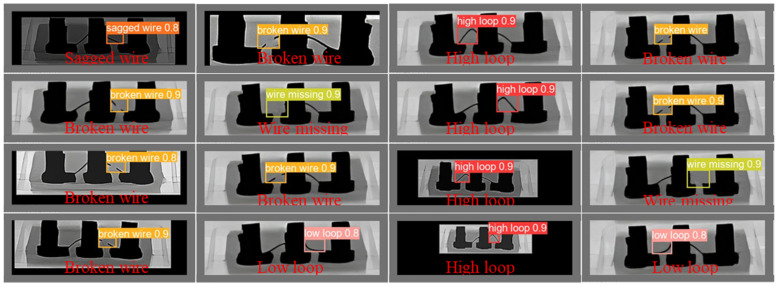
Samples of detection results of IC chip defects. Our LMNet achieved outstanding prediction on all defect classes. It shows that LMNet is capable of achieving satisfactory prediction by combing powerful contextual information and local textural features.

To facilitate the observation of the experimental data, we visualized the data in Table 5, which can be found in Figure 11. Our LMNet method achieved a $mAP_{50}$ of 99.2, outperforming all other methods. Its complexity was much lower than all classical one-level and two-level network models, with only 0.8 million parameters and 1.5 GFLOPs. Although ShuffleNetv2-YOLOv5s had the lowest GFLOPs, its detection performance was unsatisfactory, achieving only 98.4 $mAP_{50}$. At the same time, our LMNet achieved the best results in terms of detection speed, outperforming all lightweight networks, most first-level networks, and some second-level networks. Our detection speed was 1.3 times faster than that of the baseline YOLOv5n, 1.7 times faster than that of YOLOv5s, and 3.3 times faster than that of YOLOv3. At the same time, the FPS of secondary networks such as Faster-RCNN and Dynamic RCNN was less than 20, which is far from what is required for actual detection. Furthermore, their parameters and model sizes are too great for common hardware. The detection results of LMNet are shown in Figure 12. It can be seen that our LMNet could process IC chip defect images under various type and lighting conditions.

## 5. Conclusions

This paper proposes a lightweight and high-performance framework for detecting defects in IC chips using a target detection model based on convolutional neural networks. The proposed LMNet model incorporates a novel SCA module that integrates multi-scale features and adaptively assigns weights to different scales. The experimental results demonstrate that the LMNet framework achieves high prediction accuracy with small parameters and computational complexity. Specifically, LMNet achieved 99.2 $mAP_{50}$, with only a 1.7 MB model size and 1.5 GFLOPs, outperforming YOLO v5n by 0.4 points, and it had an FPS of 108.7, which is 1.7 times and 1.3 times faster than YOLO v5s and YOLO v5n, respectively. Future work will focus on optimizing the algorithm, reducing hardware costs, and applying this model to embedded platforms such as FPGA and ARM.

## Figures and Tables

**Table 1:** System parameters of X-ray device.

Tube Voltage (kV)	60–80 kV
Max tube current (mA)	0.5 mA
Focal spot size (mm)	0.5 mm
Resolution	640 × 640
Pixel size	92 µm (4.6l p/mm)
Power consumption	80 w
power supply	220 VAC
*x*-axis travel	120 mm
*y*-axis travel	120 mm
*z*-axis travel	60 mm
Repeatability	10 µ
Size	730 mm × 580 mm × 900 mm
Weight	45 kg
Control mode	Ethernet

**Table 2:** Table of dataset distribution.

Sample Dataset	High Loop Wire	Low Loop Wire	Broken Wire	Missing Wire	Sagged Wire
training set	141	243	192	409	295
validation set	21	41	15	33	50
test set	16	25	37	16	66

**Table 3:** Detailed architecture of LMNet.

Layer Name	Type	Filters	Size	Stride	Output Size
S0	Conv	16	3	1	416 × 416 × 16
S1	MaxPool	/	3	2	208 × 208 × 16
S2	RGC	32	1	1	208 × 208 × 32
S3	MaxPool	/	3	2	104 × 104 × 32
S4	RGC	64	1	1	104 × 104 × 64
S5	MaxPool	/	3	2	52 × 52 × 64
S6	RGC	64	1	1	52 × 52 × 64
S7	MaxPool	/	3	2	26 × 26 × 64
S8	RGC	128	1	1	26 × 26 × 128
S9	MaxPool	/	3	2	13 × 13 × 128
S10	RGC	256	1	1	13 × 13 × 256
S11	MaxPool	/	3	1	13 × 13 × 256
S12	RGC	512	1	1	13 × 13 × 512
S13	SCA	1024	5	/	13 × 13 × 1024
S14	Conv	512	1	1	13 × 13 × 512
S15	Conv	256	1	1	13 × 13 × 256
S16	Conv	255	1	1	13 × 13 × 255
S17	Conv	128	1	1	13 × 13 × 128
S18	Upsample	/	/	/	26 × 26 × 128
S19	Concat	/	/	/	26 × 26 × 256
S20	Conv	256	1	1	13 × 13 × 256
S21	Conv	255	1	1	13 × 13 × 255

**Table 4:** Ablation study of RGC module.

Residual	Ghost Conv	$mAP_{50}$	Params	GFLOPs (G)
		98.6	0.96	1.7
	✓	98.8	0.81	1.5
✓		99.0	0.99	1.7
✓	✓	99.2	0.81	1.5

**Table 5:** Effect comparison of different attention mechanisms in SCA module.

SE	CBAM	CA	$mAP_{50}$	Params	GFLOPs (G)
✓			98.5	1.34	1.7
	✓		99.2	0.81	1.5
		✓	98.8	0.78	1.5

**Table 6:** The recognition results compared with state-of-the-art methods.

Method	$mAP_{50}$	FPS	GFLOPs (G)	Params (M)	Size (MB)
Faster R-CNN-ResNet50	95.9	16	249.9	28.3	108
Dynamic R-CNN-ResNet50	96.4	15.8	248.5	28.7	106.9
RetinaNet-ResNet50	96.2	19.4	227.8	24.1	93.4
SSD300-VGG16	94.9	45.5	30.7	24.3	92.6
VFNet-ResNet50	95.1	15.5	224.5	26.8	98.3
YOLOv3	98.5	33.3	155.1	59.1	117
YOLOv5s	98.9	64.1	15.9	6.7	13.7
MobileNetv3-YOLOv5s	99.2	51.8	4.7	2.6	5.6
ShuffleNetv2-YOLOv5s-	98.4	66.2	1.1	0.3	0.9
GhostNet-YOLOv5s-	99.0	50.2	8.3	5.4	11.2
YOLOv5n	98.8	84.0	4.3	1.8	3.6
LMNet (ours)	99.2	108.7	1.5	0.8	1.7

## Data Availability

Data are available on reasonable request from the corresponding authors.
